# TECHNOLOGY TO SUPPORT OLDER ADULTS' HEALTH, SAFETY, AND WELLBEING

**DOI:** 10.1093/geroni/igac059.1225

**Published:** 2022-12-20

**Authors:** Walter Boot

## Abstract

Technology has revolutionized the ability to deliver interventions to support older adults’ health, safety, and wellbeing, and technology-based solutions have become increasingly important in the delivery of remote care during the COVID-19 pandemic. However, these interventions and novel approaches require a careful understanding of older adults’ needs, preferences, and abilities, and need to undergo tests of feasibility, acceptability, and efficacy. This session will present a sampling of research examining the development, testing, and implementation of technology-based solutions to support older adults. R. Azevedo will present on the development of a novel digital therapeutic for the self-management of hypertension medication adherence among older adults. S. Kwon will present on the initial efficacy of an app-based mindfulness-meditation intervention to alleviate stress and depressive symptoms among bereaved older adults. Y. Du will discuss the use of a commercial fitness tracker to facilitate activity self-monitoring among overweight diabetic older adults with and without kidney disease. S. Dimmick will discuss the development of a novel augmented reality (AR) safety checklist to reduce fall risk among older adults. Finally, F. Jain will present on the development of a new mobile application platform to meet the needs of family dementia caregivers informed by focus groups and an inductive and deductive mixed method analytic approach. Themes of the necessity of a user-centered design approach in the development of technology-solutions will be emphasized.

